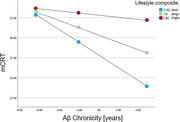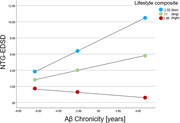# Effects of lifestyle factors on Alzheimer’s disease symptoms in adults with Down syndrome

**DOI:** 10.1002/alz.086288

**Published:** 2025-01-03

**Authors:** Emily K Schworer, Matthew D Zammit, Benjamin L Handen, Ozioma C Okonkwo, Christy L. Hom, Beau Ances, Bradley T. Christian, Sigan L Hartley

**Affiliations:** ^1^ Waisman Center, University of Wisconsin‐Madison, Madison, WI USA; ^2^ University of Pittsburgh, Pittsburgh, PA USA; ^3^ Wisconsin Alzheimer’s Disease Research Center, School of Medicine and Public Health, University of Wisconsin‐Madison, Madison, WI USA; ^4^ University of California, Irvine, Irvine, CA USA; ^5^ Department of Neurology, Washington University at St. Louis, St. Louis, MO USA

## Abstract

**Background:**

Lifetime risk for symptomatic Alzheimer’s disease (AD) in people with Down syndrome (DS) is 90%, with the age of onset of symptomatic AD ranging widely. Identifying resiliency factors related to a later age of symptoms is of critical importance for the DS community. This study investigated lifestyle factors hypothesized to moderate the association between amyloid‐beta (Aβ plaques) and AD symptoms in adults with DS.

**Method:**

Participants were 63 adults with DS (M age = 40.21 years, SD = 7.73). The majority of participants were cognitively stable (84.1%), 6.3% had dementia, and 9.6% had mild cognitive impairment. Participants completed an Aβ PET scan using [^11^C] Pittsburgh Compound‐B (PiB) and Aβ trajectories were modeled using a sampled iterative local approximation algorithm to assign each individual a chronicity value, centered at 18 Centiloids. Participants completed the modified Cued Recall Test (mCRT) and caregivers completed the National Task Group‐Early Detection Screen for Dementia (NTG‐EDSD). Caregivers were also asked to rate the amount of time the adult with DS participated in cognitive, social, and vocational activities. Adults with DS wore an Actigraph accelerometer for 7 days to assess daily step count. A lifestyle composite score was created.

**Result:**

Two moderation tests were run, with Aβ chronicity as the predictor, AD symptoms (mCRT or NTG‐EDSD) as the dependent variable, and lifestyle composite as a moderator. There was a significant interaction of lifestyle composite X chronicity on mCRT, *t* = 3.03, *p* = .004 and NTG‐EDSD, *t* = ‐3.29, *p* = .002. Participants who engaged in lifestyle activities for less time than average experienced a greater effect of chronicity on mCRT (Figure 1) and NTG‐EDSD (Figure 2).

**Conclusion:**

Lifestyle activities moderated the association between Aβ chronicity and AD symptoms. Participants who had active lifestyles with more cognitive, social, physical, and vocational activities evidenced fewer AD symptoms (i.e., less cognitive impairment) given similar Aβ chronicity values than participants who engaged in fewer activities. This study was limited to cross‐sectional associations. Future longitudinal research should tease out time‐ordered effects. Modifiable lifestyle factors may allow adults with DS to maintain cognitive functioning for longer in the face of early AD pathology.